# Framing vaccination as a collective responsibility increases intentions to vaccinate

**DOI:** 10.1177/14034948261438058

**Published:** 2026-04-19

**Authors:** Sebastian B. Bjørkheim, Tony Leino, Bjørn Sætrevik

**Affiliations:** 1 Operational Psychology Research Group, Department for Psychosocial Science, University of Bergen, Bergen, Norway; 2 Norwegian Competence Centre for Gambling and Gaming Research, Department for Psychosocial Science, University of Bergen, Bergen, Norway

**Keywords:** Responsibility, motivation, vaccination, health protective behaviour, altruism, health communication, public health

## Abstract

**Aims:**

The decision to vaccinate may be motivated by self-protection or community protection, and people may to varying degrees feel a personal responsibility or a collective obligation to vaccinate. In two preregistered survey experiments during the COVID-19 pandemic, we examined how a benefit framing (personal *vs*. collective) and a responsibility framing (personal *vs*. collective) influence vaccine intention.

**Methods:**

We used a 2×2 survey experiment design to manipulate vaccine promotion framing systematically. The experiments were conducted on large, overlapping representative samples of Norwegian adults. Separate survey experiments were performed before (*N*=5474) and after (*N*=1789) the COVID-19 vaccine rollout in Norway.

**Results:**

Both experiments showed that framing vaccination choice as a collective (*vs*. personal) responsibility increased vaccine intention. In the first experiment there was no effect of benefit framing, while in the second experiment emphasising personal (*vs*. collective) benefit increased vaccine intentions. There was no interaction between the factors in either experiment. We also observed a considerable increase in vaccine intention between the first and second experiment.

**Conclusions:**

**These findings indicate that highlighting collective responsibility may boost vaccine uptake, whereas it is more difficult to conclude about emphasising personal versus collective benefits. Message framing may be worth considering when designing health communication strategies during infectious disease outbreaks.**

## Background

The COVID-19 pandemic highlighted the importance of effective health communication in managing infectious diseases. Health authorities issued various recommendations and mandates, such as social distancing, mask-wearing, travel restrictions, isolation, and vaccination. The public’s compliance with such measures can be influenced by how they were communicated. While some measures are uncontroversial, vaccination campaigns sparked considerable debate in many countries [1]. This is noteworthy, as extensive vaccination is one of the most important measures to curb disease transmission effectively and prevent overload on hospitals.

Compliance with infection control measures is partly determined by psychological factors such as trust, personality traits, risk awareness, perceived threat and perceived benefit of taking precaution [[2–5]. At the same time, vaccine hesitancy and uptake are influenced by a broader set of psychosocial and contextual factors, including structural conditions, social norms, communication environments and health system characteristics, in addition to individual psychological antecedents such as confidence, complacency and collective responsibility [6–8]. An important dimension within these determinants concerns individuals’ motivational orientation towards vaccination. Some individuals are primarily motivated by personal health benefits, whereas others are more motivated by the broader societal impact of vaccination [2,9,10]. These motivational differences are particularly relevant because they may be influenced by how responsibilities and benefits are framed in public health communications.

### Personal and collective rationales for vaccination

When deciding on whether to vaccinate, people may consider both individual and collective factors, as vaccination protects oneself and contributes to the health of the community. According to the health belief model, individuals base their decisions on the perceived susceptibility, severity of the disease, and the benefits and barriers of taking precautions [11,12]. The protection motivation theory further emphasises how people are motivated to safeguard their personal health through protective actions [13,14]. While personal health concerns motivate some individuals to vaccinate, others are motivated by the broader societal impact of their decisions [9,15,16]. This distinction between advantages for the individual and for the society connects to Rose’s argument that prevention must be judged both for ‘sick individuals’ and ‘sick populations’, where a small change in individual risk can translate into a large benefit at the population level, but may seem less compelling from a strictly individual point of view [17]. Prosocial motivation, where individuals consider the wellbeing of others, may play a key role in vaccine uptake [18]. This may be because people recognise that vaccination helps protect vulnerable groups and contributes to herd immunity. For example, Arnesen and colleagues [15] tested whether emphasising collective benefits versus individual benefits of vaccination would influence people’s intention to vaccinate. They found that both benefit frames increased vaccination intentions, but emphasising collective benefits had a stronger effect [15]. Related research on COVID-19 vaccination shows that individuals with higher interpersonal and political trust are more likely to vaccinate [10,19], which suggests that collective concerns influence vaccine uptake.

Personal responsibility may also influence vaccination decisions [20]. Personal responsibility describes the idea that vaccination is primarily an individual decision and obligation, grounded in one’s own judgment about what a responsible person should do in this situation. By contrast, collective responsibility refers to a shared civic obligation to contribute to public health by getting vaccinated. This is related to the 5C model’s definition of collective responsibility as people’s intention to protect others by their own vaccination [6,8]. These concepts differ in the locus of obligation in that personal responsibility emphasises individual choice and self-governance, whereas collective responsibility emphasises one’s role as a member of a community and the expectation to act for the common good [6,21]. Individuals who feel a strong responsibility towards public health may be more inclined to get vaccinated [22]. Feelings of personal obligation and perceived control are key predictors of behavioural intentions in social-cognition models, and people who view vaccination as part of being a responsible person are more likely to intend to vaccinate [21,23]. This sense of responsibility is shaped by internalised values, moral self-concept, and perceived personal obligation, and may be indirectly influenced by social norms to the extent that such norms are internalised rather than experienced as external expectations [20]. At the same time, studies using the 5C model show that collective responsibility is a strong and consistent correlate of vaccination intentions [6]. Similarly, it has been shown that people who view vaccination as a civic duty and a contribution to the greater good are generally more likely to vaccinate [24]. Conversely, those who prioritise personal freedom or have lower trust in authorities may be less likely to view vaccination as a responsibility, which is associated with lower vaccination rates [1,10].

Public health communication often highlights the benefits of compliance with infection control measures by focusing on positive outcomes (benefit) for the individual. There have been mixed results regarding the effectiveness of appeals to personal versus collective benefits. On the one hand, messages that emphasise collective benefits have generally been found to be more successful in promoting adherence to infection control measures [25–28]. On the other hand, self-preservation and protecting one’s closest family are also strong motivators for complying with infection control measures. Emphasising personal and familial protection was shown to have a greater impact on increasing social distancing intentions than appeals focused on protecting society at large [29]. While ‘protecting one’s family’ may be seen as a personal benefit, this motive can also be seen as prosocial and closely related to collective benefits, as it concerns reducing risk for close others rather than only for oneself. Messages focused on personal benefits have also been found to be more effective in promoting vaccination, especially among individuals who perceived a high risk of illness [30]. Individuals who believed they were at high risk of contracting COVID-19 were more likely to engage in protective behaviours when messages mentioned personal consequences of inaction [31]. Similarly, emphasising the direct health benefits of vaccination, such as preventing severe illness or death, increased vaccine uptake among people more afraid of the disease [9]. Taken together, these findings suggest that personal and collective motivations are not mutually exclusive. While self-protection may be especially salient under conditions of high perceived risk, both personal and collective considerations can meaningfully influence vaccination decisions, depending on individual characteristics and contextual factors.

In this study we operationalise vaccination intention as the self-reported willingness to accept an offered vaccine. Vaccination intention should therefore be understood as only one specific component within this broader construct of vaccine hesitancy, and we specifically focus on how framing vaccination choice along dimensions of responsibility and benefit influence vaccination intention in the context of a COVID-19-like vaccine. The World Health Organization (WHO) described vaccine hesitancy as a broader motivational state of being conflicted about, or opposed to, getting vaccinated, which includes intentions and willingness to vaccinate [32]. Meta-analytic reviews have shown that intentions predict later health behaviour with medium to large effects [33,34]. A review of vaccination studies showed a comparable association, with a medium to high correlation between intention and subsequent uptake [35].

### Aims

While extensive research has explored the motivations for vaccination and vaccine hesitancy, a gap remains in understanding how personal and collective considerations interact to influence vaccination decisions. Both personal and collective benefit messages increased vaccination intentions in Norway, with collective benefits having a stronger effect [15]. To our knowledge, no studies have explored how framing vaccination in terms of personal versus collective benefits interacts with appeals to personal versus collective responsibility. Understanding these dynamics will provide insight into the relative influence of each factor and can uncover potential interaction effects between them. This can help public health authorities tailor messages to address both personal and collective motivations.

### Current study

The present study examined framing effects on intentions to take a new vaccine against a disease such as COVID-19 during the COVID-19 pandemic in Norway, rather than on vaccine intentions in general. We fielded two online survey experiments to nationally representative samples of Norwegian adults. The first experiment was conducted when numerous infection control measures were in place, but before vaccines against COVID-19 were available. By the time of the second data collection, approximately 90% of the adult Norwegian population had received at least one dose of the COVID-19 vaccine, and no infection control measures were active. We used a 2×2 between-subjects design, with experimental conditions that varied based on two factors: the benefits of vaccination (personal or collective) and the responsibility for getting vaccinated (personal or collective).

### Hypotheses and preregistrations

Hypotheses were preregistered before data from experiment 1 was available to the researchers (see https://osf.io/p2xt5) and identical hypotheses were preregistered before experiment 2 data was available (see https://osf.io/x5fc4).

Numerous findings, including one using a similar approach in Norway, have suggested that prosocial framing is often more effective than purely self-focused framing in increasing compliance with health-protective measures [15,26–28]. In particular, Arnesen and colleagues [15] found that emphasising collective benefits of vaccination (herd protection) increased vaccination intentions more than emphasising personal benefits. In a pandemic context, where protecting others and ‘doing one’s part’ were highly salient, we therefore hypothesised (H1) that participants would show greater intention to get vaccinated when messages emphasise collective benefits rather than personal benefits (a main effect for appeals to collective benefits), while recognising that prior findings on personal versus collective benefit messages are mixed.

Based on previous findings and established health-behaviour models, we also expected responsibility cues to matter for vaccination decisions. Feelings of personal obligation and perceived control are important predictors of intention in social-cognition models, and people who see vaccination as part of ‘being a responsible person’ are more likely to intend to vaccinate [21,23]. At the same time, research using the 5C model has shown that collective responsibility is a robust correlate of vaccination intentions [6]. Our preregistered hypothesis (H2) focused on self-focused responsibility. Specifically, we expected that participants would indicate a greater intention to get vaccinated when the message emphasises personal responsibility (i.e. ‘you should choose to vaccinate’) rather than collective responsibility (i.e. ‘as many people as possible should vaccinate’), because framing vaccination as a personal obligation may strengthen perceived responsibility and commitment to act. Based on this, we hypothesised (H2) that participants would indicate a greater intention to get vaccinated when the message emphasises the personal responsibility rather than collective responsibility (a main effect for personal responsibility). Thus, H2 tested whether this self-focused obligation cue would have an advantage.

We further anticipated that benefit and responsibility cues might interact. Conceptually, benefit framing primarily targets expectations about outcomes (what vaccination achieves), whereas responsibility framing targets a sense of obligation (what one ought to do). If these pathways are partly independent, combining a communal outcome (‘collective benefits’) with a self-focused obligation cue (‘personal responsibility’) could be especially motivating, because it links doing one’s part personally to a clear benefit for the wider community [6,21]. We therefore hypothesised (H3) that collective-benefit framing would have a greater effect when combined with personal responsibility framing than with collective responsibility framing. Thus, H3 tested whether the combination of a collective outcome and individual obligation would yield higher intentions than would be expected from the main effects alone.

## Methods

### Data collection

The Norwegian Citizen Panel was used to collect the data (see https://www.uib.no/en/citizen for details). Experiment 1 was collected as part of an additional ‘fast track’ data collection round in 2020, specifically introduced to address COVID-19-related issues. The field period lasted for 8 days between 26 August and 2 September in 2020 (see methodology report: https://osf.io/5h2sb). At this time the COVID-19 pandemic was between its first and second wave of high infection spread in Norway. A number of infection control measures were in place at that time. Several vaccine candidates were being examined at the time, but no conclusive reports of success had been reported, and the projected timeline for distributing a vaccine was in about half a year.

The data collection for experiment 2 was executed similarly to experiment 1 (see full methodology report on https://osf.io/jyzat), between 31 October and 28 November 2022. At this time, no infection control measures against COVID-19 were in place, but the public was encouraged to be mindful of pulmonary symptoms and take precautions if necessary. Vaccines against COVID-19 had been rolled out nationwide and around 90% of the adult population had received at least one dose of the COVID-19 vaccine at this point (see https://www.fhi.no/en/id/corona/coronavirus-immunisation-programme/ for more information).

Although the Norwegian Citizen Panel is longitudinal, the two waves are analysed as repeated cross-sectional design, consistent with our preregistration. There are three reasons for this. First, our aim was to compare framing effects for responsibility and benefit at two distinct stages of the pandemic, before and after vaccine roll out, rather than to track individual change over time. We thus do not have any preregistered hypotheses for tracking individual changes over time. Second, the vignette wording and the broader pandemic context differed between waves, so within-person comparisons would blend message effects with contextual shifts. Third, panel attrition and replenishment would yield a smaller matched sample and reduce statistical power without offering clear analytical benefit.

### Participants

The experiments had a representative sample of Norwegian adults aged 18 years and older (based on random selection from the Norwegian Tax Registry). The experiment 1 survey was completed by 5531 participants (response rate 81.8%). Recruitment to experiment 2 was smaller, and 1789 participants took part (response rate 77%). The sample deviated slightly from population statistics in terms of age, education level and place of residence (see full methodology report for the first data collection here: https://osf.io/2u3dp and the second here https://osf.io/jyzat). Younger adults (18–29 years) and middle-aged adults (30–59 years) are underrepresented in this sample, while older adults (60 years and older) are slightly overrepresented. Similarly, individuals with lower education levels are underrepresented, while those with university education are overrepresented.

Experiment 1 (*N*=5478) included 49% men and 51% women. Around two thirds reported university or college education (about 63%), about 30% had upper secondary education, and about 5% had elementary education or less; 46% were born in 1959 or earlier, 48% in 1960–1989, and about 7% in 1990 or later. Experiment 2 (*N*=1793) had a similar gender balance (about 52% men and 48% women) and slightly higher educational attainment, with about 66% reporting university or college education, 29% upper secondary, and 4% elementary or less. In experiment 2 about 52% were born in 1959 or earlier, 44% in 1960–1989, and 4% in 1990 or later.

### Materials and variables

Participants read a brief introduction asking them to imagine that the health authorities offered a vaccine against COVID-19 which had not been tested for long-term side-effects. The four randomly assigned conditions consisted of an introductory scenario description, a sentence containing the benefit manipulation, and a sentence containing the responsibility manipulation. See materials and codebook for experiment 1 online (https://osf.io/5h2sb).

The benefit manipulation sentence emphasised either an individual benefit or a collective benefit from taking the vaccine. The individual benefit was phrased as (translated from Norwegian): ‘The vaccine provides an effective protection against the disease and will help you stay healthy’, whereas the collective benefit sentence read: ‘Some will be unable to take the vaccine for medical reasons, and in order to prevent them from becoming infected, as many people as possible must take the vaccine.’

The responsibility manipulation sentence emphasised either individual responsibility (emphasising personal choice) or collective responsibility (emphasising majority action) for taking the vaccine. Individual responsibility was phrased as: ‘It is therefore an advantage if individuals like you are vaccinated’, whereas collective responsibility read: ‘It is therefore important that as many people as possible in society are vaccinated.’ Thus, the responsibility conditions also differed in tone, with the collective responsibility frame using a more prescriptive wording (‘important’) than the personal responsibility frame (‘advantage’).

The outcome variable was a response to the probe ‘How sure are you that you would get vaccinated if you are offered to take the vaccine?’ measured on a five-point Likert-type item with options: (1) ‘I am completely certain that I would not take the vaccine’; (2) ‘I would probably not take the vaccine’; (3) ‘I am not sure if I would take the vaccine’; (4) ‘I would probably take the vaccine’; (5) ‘I am completely certain that I would take the vaccine’.

Compared with the vignette used in experiment 1, the vignette used in experiment 2 removed mention of the untested long-term side-effects as this was considered to be less relevant later in the pandemic. Instead, we highlighted the practical drawbacks of booking an appointment with the GP to take the vaccine. These contextual changes were necessary for realism but may have introduced additional variance beyond the framing manipulation itself. As our hypothesis tests compare the framing effect of different versions of the communication within each time point, the contextual differences between the vignettes are unlikely to affect the framing comparisons. However, these changes may influence overall vaccination intentions and should be considered when interpreting differences between the two time points.

### Research needs from experiment 1

Motivations for vaccination may have changed when the public became more familiar with the pandemic, and vaccines against COVID-19 had been rolled out in Norway. Given the significant changes in the pandemic trajectory, it is valuable to re-examine whether collective or individualistic appeals impact vaccination intentions. To ensure consistency and clarity in testing our research questions, we did not change experiment 2 hypotheses based on experiment 1 results. Experiment 2 updated the framing to be more context-specific, using standardised wording (e.g. ‘an advantage’ in all conditions) and referencing a new vaccine ‘against diseases like COVID-19’. Note that the conditions in experiment 1 also differed in directive tone (‘will help you stay healthy’ in the personal-benefit condition *vs*. ‘. . .as many people as possible must take the vaccine’ in the collective-benefit condition). Experiment 2 therefore standardised the tone by using ‘an advantage’ in every condition. The benefit manipulation in experiment 2 again emphasised either an individual or a collective benefit. In the individual benefit conditions, the text stated that ‘Those who take the vaccine will stay healthy more easily, and will be able to live more normally during a pandemic outbreak.’ In the collective benefit conditions, the text stated that ‘If many people take the vaccine, a pandemic outbreak will have less negative consequences for society.’ The responsibility manipulation emphasised either personal or collective responsibility. In the personal responsibility conditions, the text ended with ‘It is therefore an advantage that you choose to vaccinate yourself.’ In the collective responsibility conditions, the text ended with ‘It is therefore an advantage that as many people as possible in society choose to be vaccinated.’

The overall changes make the wording more context specific and highlight the practical benefits of vaccination during a pandemic. See full materials and codebook for experiment 2 online (https://osf.io/mn54a).

## Results

### Overall intention to vaccinate

Across all experimental conditions, participants exhibited an inclination to accept the vaccine, with more individuals expressing positive intentions than reluctance. Comparing the results from experiment 1 (September 2020) with experiment 2 (November 2022), we observed a substantial increase in vaccine intentions over time (see Table I). Descriptive analysis (see Tables II and III) revealed consistent demographic patterns across the two survey waves. In experiment 1 (collected in 2020) the highest mean intention scores appeared among respondents born in 1959 or earlier (M=3.49, SD=1.10), whereas the youngest cohort, born in 1990 or later, showed relatively less intention M=3.37 (SD=1.10). We also observed a higher intention to vaccinate among those with a university or college education (M=3.43, SD=1.08) compared with participants with upper secondary education who scored M=3.31 (SD=1.17) and those with elementary schooling who scored M=3.29 (SD=1.18). Gender differences were minimal, with men at M=3.44 and women at M=3.33 (difference 0.11). Experiment 2 displayed the same rank order with higher overall means.

**Table I.** Number of responses and percentage share for each response option across both experiments.

Response option	*N* (Experiment 1)	% (Experiment 1)	*N* (Experiment 2)	% (Experiment 2)
I am completely certain that I would not	391	7.14%	63	3.51%
I would probably not take the vaccine	698	12.74%	90	5.02%
I am not sure if I would take the vaccine	1695	30.94%	168	9.37%
I would probably take the vaccine	1787	32.62%	521	29.06%
I am completely certain that I would	907	16.56%	951	53.04%

**Table II.** Experiment 1 (2020): Vaccination intention by demographic group.

Group	*n*	M (SD)
Sex/gender		
Male	2678	3.44 (1.15)
Female	2800	3.33 (1.08)
Education		
No education/elementary	261	3.29 (1.18)
Upper secondary	1661	3.31 (1.17)
University/college	3445	3.43 (1.08)
Birth cohort		
Born 1959 or earlier	2496	3.49 (1.10)
Born 1960–1989	2604	3.29 (1.13)
Born 1990 or later	378	3.37 (1.10)

**Table III.** Experiment 2 (2022): Vaccination intention by demographic group.

Group	*n*	M (SD)
Sex/gender		
Male	940	4.26 (1.04)
Female	853	4.20 (1.04)
Education		
No education/elementary	76	4.16 (1.08)
Upper secondary	511	4.15 (1.11)
University/college	1189	4.26 (1.01)
Birth cohort		
Born 1959 or earlier	930	4.46 (0.89)
Born 1960–1989	786	4.02 (1.14)
Born 1990 or later	77	3.68 (1.12)

In December 2021 the citizen panel asked about the panel members’ COVID-19 vaccination status. Descriptive results showed a very high uptake (95.7% of at least two doses) which is higher than the national registry figure of approximately 90% for that period (see Table IV).

**Table IV.** Response distribution for COVID-19 vaccination (self-reported) behaviour in the Norwegian Citizen Panel measured in December 2021.

Response category	Frequency	Percentage
Yes, I have been vaccinated (fully vaccinated, two doses) and will take a third dose if offered	1139	88.9%
Yes, I have been vaccinated (fully vaccinated, two doses), but will not take a third dose if offered	87	6.8%
Yes, I have been vaccinated (part-vaccinated, one dose) and will take a second dose	7	0.5%
Yes, I have been vaccinated (part-vaccinated, one dose), but will not take a second dose	2	0.2%
No, I haven’t been vaccinated, but do intend to be	5	0.4%
No, I haven’t been vaccinated and do not intend to be	28	2.2%
No, I haven’t been vaccinated and cannot be vaccinated for various reasons	9	0.7%
Not answered	4	0.3%

Responses to the question: I will be (or have been) vaccinated against coronavirus.

### Confirmatory analyses experiment 1

We tested the preregistered hypotheses using two-way analyses of variance (ANOVA) with benefit framing (personal *vs*. collective) and responsibility framing (personal *vs*. collective) as between-subjects factors, separately for each experiment. For H1, we compared mean vaccination intentions in the personal-benefit conditions with the collective-benefit conditions, collapsing across the two responsibility levels. For H2, we compared mean intentions in the personal-responsibility conditions with the collective-responsibility conditions, collapsing across the two benefit levels. For H3, we examined the benefit by responsibility interaction term to assess whether the effect of benefit framing differed by responsibility framing.

### No effect of collective benefit

The H1 hypothesis anticipated higher intention to vaccinate when the scenario emphasised collective benefits (M=3.36, SD=1.13), as opposed to personal benefits (M=3.42, SD=1.11). A two-way ANOVA did not reach the cut-off for significance for an effect between the benefits conditions (*F* [1,5474]=3.62, *P*=0.057).

### Non-predicted effect of collective responsibility

The H2 hypothesis anticipated higher intention to vaccinate when the scenario appealed to the personal responsibility (M=3.35, SD=1.10), as opposed to collective responsibility (M=3.43, SD=1.14). A two-way ANOVA found a significant main effect (*F* [1,5474]=7.29, *P*=0.007, η2=0.001) in the opposite direction, such that participants were more willing to vaccinate when the scenario emphasised collective responsibility. According to conventional interpretations [36], the responsibility factor explained a very small proportion of the observed variance in intention to take the vaccine.

### No interaction effect between benefit and responsibility factor

The H3 hypothesis anticipated an interaction effect between ‘collective benefits’ and ‘personal responsibility’, such that there would be higher intention to vaccinate when both collective benefits and personal responsibility was emphasised. A two-way ANOVA did not reach the cut-off for significance for an interaction effect between the two factors (*F* [1,5474]=3.59, *P*=0.058).

### Confirmatory analyses experiment 2

#### Non-predicted effect of personal benefit

The H1 hypothesis anticipated higher intention to vaccinate when the scenario emphasised collective benefits (M=4.30, SD=1.03), as opposed to personal benefits (M=4.16, SD=1.05). A two-way ANOVA found a statistically significant and very small effect in the opposite direction of H1 (*F* [1, 1789]=8.28, *P*=0.004, η2=0.005), indicating that appeals to personal benefit increased vaccination intentions.

#### Non-predicted effect of collective responsibility

The H2 hypothesis anticipated higher intention to vaccinate when the scenario appealed to the personal responsibility (M=4.18, SD=1.08), as opposed to collective responsibility (M=4.29, SD=0.99). A two-way ANOVA showed a very small significant main effect (*F* [1, 1789]=5.15, *P*=0.023, η^2^ =0.003) in the opposite direction, indicating that appeals to collective responsibility increased vaccination intentions. This replicated the main effect of collective responsibility from experiment 1.

#### No interaction effect between benefit and responsibility factor

The H3 hypothesis anticipated an interaction effect between ‘collective benefits’ and ‘personal responsibility’, such that vaccine intention was greater in the collective benefits condition that also emphasised personal responsibility. A two-way ANOVA did not find an interaction effect between these factors (*F* [1, 1789]=1.13, *P*=0.28).

## Discussion

The current study examined how vaccination intentions were influenced by framing vaccination choice as a personal or collective responsibility, and by emphasising personal or collective benefits. Our findings show that emphasising collective responsibility rather than personal responsibility consistently increased participants’ intention to vaccinate, albeit with small effect sizes. This aligns with evidence that suggests that collective considerations promote vaccination behaviour [9,18]. It is worth noting that this effect may be particularly pronounced in societies with high levels of social cohesion and trust, such as the Scandinavian context in which this study was conducted [15,37]. The value placed on communal wellbeing may make appeal to collective responsibility a more effective strategy in such settings. However, the collective responsibility message used wording that may have implied that ‘most Norwegians intend to vaccinate’, that could prompt participants to conform to a majority norm rather than acting from genuine concern for the collective. Disentangling these alternative explanations may require future work that manipulates normative information independently of collective benefit cues and controls for responding to social desirability. Also, while the modest effect size indicates that collective responsibility cues matter, it is likely one of many influences on vaccination decisions rather than the dominant driver.

The test of benefit framing yielded more varied results. There was no difference between emphasising personal versus collective benefits in experiment 1, which suggests that either frame was equally persuasive in that early stage of the pandemic. However, in experiment 2 (after COVID-19 vaccination had begun), personal benefit framing had a slight advantage over collective benefit framing. This suggests that despite the prosocial nature of vaccination, individuals may prioritise personal health outcomes when deciding on whether to vaccinate. This finding is broadly consistent with the core concepts of protection motivation theory [13,14], and with research indicating that personal risk perception strongly motivates the adoption of protective behaviours [30]. However, the fact that personal benefit did not show an effect in experiment 1 but did so in experiment 2 should be interpreted with caution. The benefit items were reworded between the two experiments and were administered in different phases of the pandemic, so we cannot determine whether the apparent personal-benefit effect in experiment 2 reflects the manipulation, revised wording, the changed context, or an effect between these. These cross-experiment contrasts are therefore descriptive rather than strong evidence for a change in the underlying framing effect.

Interestingly, our findings contrast with pre-COVID-19 research on Norwegian vaccination behaviour. A study on vaccine framing in Norway [15] found that both personal benefit and collective benefit framing increased vaccination intentions, but messages about collective benefits had a considerably stronger effect. In contrast, we found no difference between the factors before vaccine rollout, and a stronger effect for personal benefit after vaccine rollout. This could be due to shifts in public attitudes during the COVID-19 pandemic. Because of widespread media coverage, personal experiences with the virus, and prolonged uncertainty, individuals may have felt a stronger need to prioritise self-protection. This is consistent with protection motivation theory’s emphasis on personal threat appraisal [13,14]. It is worth noting that for behaviours such as vaccination, personal protection and concern for others are often intertwined, making it difficult to draw a clear distinction between self-oriented and community-oriented motivations. This ambiguity also extends to the role of family and close relations, which may blur these categories further.

There was no support for our expectation that a framing of collective benefits combined with personal responsibility would be more effective. Across both experiments, the absence of a reliable interaction effect between benefit and responsibility suggests that outcome-focused and duty-focused cues operated largely independently under our stimuli and context. This is noteworthy given that many public health messages implicitly combine both communal duty and individual gain. Our results indicate that while both factors can influence intentions, their effects may be largely independent from each other, and context dependent in a pandemic cycle. This finding contributes to ongoing debates about whether and how multiple message frames interact [9,24,26,27].

We observed small effect sizes overall. This may be unsurprising given the subtle framing manipulations in the current study. Nevertheless, it is important to note that the effect sizes are smaller than in similar studies [15,26]. Even so, small yet reliable effects from preregistered studies on representative samples can provide meaningful information. Slight changes to communication can influence many people if the message reaches a large audience, and even slight reductions in infection rates can have a substantial impact on public health due to the exponential spread of diseases like COVID-19 [38]. Moreover, refining a key message may prove to be a low-cost way to improve health outcomes. Interestingly, our data showed that there was a considerable increase in vaccination intentions during the COVID-19 pandemic. This indicate that pandemic events and familiarity with vaccination campaigns may have a substantial effect on people’s vaccination intention, but our design does not permit attribution of this change to specific contextual features. This is particularly relevant in the context of ongoing public health crises, in which sustained communication efforts are necessary to maintain and enhance public compliance with infection control measures.

### Limitations

A key limitation for this kind of research is that it measures stated intentions to vaccinate, rather than observing actual vaccination behaviour. Our experimental paradigm does not provide multi-item measures of the constructs, so they should be seen as targeted manipulations of perceived benefit and responsibility rather than as direct operationalisations of the validated subscales [6,39]. Consequently, the extent to which the observed effects in our study translate into actual vaccination uptake remains unclear. Moreover, research shows that changes in intentions often overestimate subsequent changes in behaviour [40].

It is important to note that the responses in experiment 2 were inclined towards a high level of intention to vaccinate, and this left limited room to show the full extent of any positive impact of the manipulations. This might have contributed to the relatively small effect sizes observed in this study. Vaccination intentions close to ceiling level suggest that motivational barriers were already perceived as low by the Norwegian sample. Further gains are thus likely to come from practical or access-oriented interventions rather than additional persuasion. By contrast, in settings where baseline intention is lower and more variable, the same framings could yield proportionally larger public health benefits. It is thus important to keep in mind the need to tailor communication strategies to local conditions.

Also, in experiment 1 our ‘benefit’ and ‘responsibility’ manipulations unintentionally coupled the focus of benefit and responsibility with a shift from descriptive wording to a more prescriptive tone, introducing a potential confound that may partly account for the null benefit effect and the small but reliable responsibility effect. A further limitation is that the benefit and responsibility items were modified between experiment 1 and experiment 2 and were administered in different pandemic phases, which reduces the comparability of the two experiments. As a result, differences in effects across experiments may reflect the manipulation, revised wording, the changed context, or an interaction effect between these. One should thus be careful not to overinterpret the difference in results from experiment 1 to experiment 2 as evidence of a change in the impact of personal benefit framing. Another measurement limitation is that the personal benefit sentence in experiment 1 explicitly stated that the vaccine provides ‘effective protection’, whereas the collective benefit sentence did not. As perceived effectiveness is an important determinant of vaccine confidence [6], this wording difference may have introduced a perceived effectiveness cue in addition to the intended focus on personal versus collective outcomes. Although the wording was made more parallel in experiment 2, differences between the two experiments in both item wording and pandemic context mean that cross-experiment differences in benefit effects should be interpreted with caution. In experiment 2, the ‘personal benefit’ frame emphasised that vaccinated individuals could ‘live more normally during a pandemic outbreak’. This wording may also imply broader societal benefits such as fewer restrictions, so it may not represent a purely self-focused benefit, and the observed effect should therefore be interpreted as reflecting a mix of personal and collective considerations rather than a clean personal-benefit manipulation. A related limitation concerns the responsibility manipulation in experiment 1. The collective responsibility sentence (‘it is therefore important. . .’) used a stronger prescriptive tone than the personal responsibility sentence (‘it is therefore an advantage. . .’), so differences between these conditions may partly reflect stylistic tone rather than responsibility alone. Moreover, the personal responsibility item (‘it is therefore an advantage if individuals like you are vaccinated’) may be interpreted more as an optional benefit than as a clear obligation.

It should also be noted that the vignettes within each experiment differed (see Tables V and VI). Specifically, both vignettes provided some potential drawback to taking the vaccine: Experiment 1 primed potential long-term side-effects of not taking the vaccine, whereas experiment 2 primed ease of access (‘contact your GP’). Any between-experiment difference therefore reflects these two contextual cues in addition to, or instead of, the contextual factors changing between 2020 (experiment 1) and 2022 (experiment 2). Contextual moderators were not incorporated into the experimental design of this study. Consequently, the experiment estimates average framing effects within the context at each wave, and it cannot identify which contextual features drive between-wave differences. Although we analysed the two waves as repeated cross-sections, some participants may have appeared in both experiments, which could introduce familiarity effects. Randomisation within each wave should preserve unbiased treatment comparisons, but any overlap might impact baseline intention levels and is worth considering when interpreting the results.

**Table V.** Full vignette texts for experiment 1 conditions.

Condition	Full vignette text
Personal benefit, personal responsibility	Imagine that the health authorities offer a vaccine against COVID-19 that has not been tested for long-term side-effects. The vaccine provides an effective protection against the disease and will help you stay healthy. It is therefore an advantage if individuals like you are vaccinated.
Personal benefit, collective responsibility	Imagine that the health authorities offer a vaccine against COVID-19 that has not been tested for long-term side-effects. The vaccine provides an effective protection against the disease and will help you stay healthy. It is therefore important that as many people as possible in society are vaccinated.
Collective benefit, personal responsibility	Imagine that the health authorities offer a vaccine against COVID-19 that has not been tested for long-term side-effects. Some will be unable to take the vaccine for medical reasons, and in order to prevent them from becoming infected, as many people as possible must take the vaccine. It is therefore an advantage if individuals like you are vaccinated.
Collective benefit, collective responsibility	Imagine that the health authorities offer a vaccine against COVID-19 that has not been tested for long-term side-effects. Some will be unable to take the vaccine for medical reasons, and in order to prevent them from becoming infected, as many people as possible must take the vaccine. It is therefore important that as many people as possible in society are vaccinated.

**Table VI.** Full vignette texts for experiment 2 conditions.

Condition	Full vignette text
Personal benefit, personal responsibility	Imagine that a new vaccine against diseases such as COVID-19 is offered. Those who take the vaccine will stay healthy more easily, and will be able to live more normally during a pandemic outbreak. It is therefore an advantage that you choose to vaccinate yourself.
Personal benefit, collective responsibility	Imagine that a new vaccine against diseases such as COVID-19 is offered. Those who take the vaccine will stay healthy more easily, and will be able to live more normally during a pandemic outbreak. It is therefore an advantage that as many as possible in society choose to be vaccinated.
Collective benefit, personal responsibility	Imagine that a new vaccine against diseases such as COVID-19 is offered. If many people take the vaccine, a pandemic outbreak will have fewer negative consequences for society. It is therefore an advantage that you choose to vaccinate yourself.
Collective benefit, collective responsibility	Imagine that a new vaccine against diseases such as COVID-19 is offered. If many people take the vaccine, a pandemic outbreak will have fewer negative consequences for society. It is therefore an advantage that as many people as possible in society choose to be vaccinated.

Our findings should be interpreted in light of this specific Norwegian COVID-19 context and a hypothetical new COVID-like vaccine, as vaccine attitudes are complex and context specific, varying across time, place and vaccines [7]. These considerations are important to keep in mind when extending the findings of this study to other settings. Importantly, the modest effect sizes also indicate that other factors, beyond responsibility and benefit framing, influence peoples’ intention to vaccinate.

## Conclusions

Collective responsibility framing (*vs*. personal responsibility) showed a consistent positive effect on vaccination intention across both experiments. Personal benefit (*vs*. collective benefit) showed a small positive effect in one of the experiments. The lack of an interaction effect suggests that these factors function independently, each influencing the vaccination decisions through separate mechanisms. This has implications for public health messaging, indicating that strategies can target both personal and collective motivations without expecting their combination to produce conflicting or synergistic effects. The data did not confirm our preregistered expectations, which further underscores the complexity of designing effective public health messages and highlights the unclear patterns in this research field.
